# Geography and health: role of human translocation and access to care

**DOI:** 10.1186/s40249-024-01205-4

**Published:** 2024-05-23

**Authors:** Norbert Brattig, Robert Bergquist, Danielle Vienneau, Xiao-Nong Zhou

**Affiliations:** 1 Bernhard Nocht Institute for Tropical Medicine, Hamburg, Germany; 2 Geospatial Health, Ingerod, formerly UNICEF/UNDP/World Bank/WHO Special Programme for Research and Training in Tropical Diseases (TDR), Brastad, Sweden; 3 Swiss Tropical and Public Health Institute, Allschwil, Switzerland; 4 University of Basel, Basel, Switzerland; 5 School of Global Health, Chinese Center for Tropical Diseases Research, Shanghai Jiao Tong University of Medicine, National Institute of Parasitic Diseases at Chinese Center for Disease Control and Prevention (Chinese Center for Tropical Diseases Research); NHC Key Laboratory of Parasite and Vector Biology; WHO Collaborating Centre for Tropical Diseases, Shanghai, People’s Republic of China; 6 Hainan Center for Tropical Diseases Research (Hainan Sub-Center of Chinese Center for Tropical Diseases Research), Haikou, People’s Republic of China

**Keywords:** Climate change, Emerging diseases, Re-emerging diseases, Evolution of pathogens, Geography, Human mobility, Infectious diseases, Pathogen transmission, Spatial analysis, Translocation

## Abstract

Natural, geographical barriers have historically limited the spread of communicable diseases. This is no longer the case in today’s interconnected world, paired with its unprecedented environmental and climate change, emphasising the intersection of evolutionary biology, epidemiology and geography (*i.e.* biogeography). A total of 14 articles of the special issue entitled “Geography and health: role of human translocation and access to care” document enhanced disease transmission of diseases, such as malaria, leishmaniasis, schistosomiasis, COVID-19 (Severe acute respiratory syndrome corona 2) and Oropouche fever in spite of spatiotemporal surveillance. High-resolution satellite images can be used to understand spatial distributions of transmission risks and disease spread and to highlight the major avenue increasing the incidence and geographic range of zoonoses represented by spill-over transmission of coronaviruses from bats to pigs or civets. Climate change and globalization have increased the spread and establishment of invasive mosquitoes in non-tropical areas leading to emerging outbreaks of infections warranting improved physical, chemical and biological vector control strategies. The translocation of pathogens and their vectors is closely connected with human mobility, migration and the global transport of goods. Other contributing factors are deforestation with urbanization encroaching into wildlife zones. The destruction of natural ecosystems, coupled with low income and socioeconomic status, increase transmission probability of neglected tropical and zoonotic diseases. The articles in this special issue document emerging or re-emerging diseases and surveillance of fever symptoms. Health equity is intricately connected to accessibility to health care and the targeting of healthcare resources, necessitating a spatial approach. Public health comprises successful disease management integrating spatial surveillance systems, including access to sanitation facilities. Antimicrobial resistance caused, e.g. by increased use of antibiotics in health, agriculture and aquaculture, or acquisition of resistance genes, can be spread by horizontal gene transfer. This editorial reviews the key findings of this 14-article special issue, identifies important gaps relevant to our interconnected world and makes a number of specific recommendations to mitigate the transmission risks of infectious diseases in the post-COVID-19 pandemic era.

## Background

Ecological conditions remain an important limiting factor for many infections, but our highly interconnected world together with climate change support the spread of communicable diseases across natural, geographical barriers that historically have played a strong limiting role. This has led to ‘leaking ecological niches’ such that human kind is increasingly faced with global changes with long-term consequences for biological diversity, particularly with respect to vectors and carrier animals that contribute to rapid spread of pathogens. Contributing factors are deforestation with urbanization encroaching into wildlife zones, human migration dynamics and the increase of long-distance travel. Environmental factors produce specific, ecological niches governing growth and transmission of both fauna and flora [1, 2]. The BioClim and the WorldClim (http://worldclim.org/) datasets have been used in ecological and biogeographical research to study the relationships between climate and the distribution of organisms [3, 4]. The BioClim concept represents the first species distribution modelling package and its association with the WorldClim, a high spatial resolution (> 1 km^2^) created a worldwide source of global weather and climate.

Emphasis on the presence, rather than absence, of disease has dominated medical thinking since ancient times, with the term public health first appearing in the early 1900s [5]. The paradigm-shift from the idea of ‘miasmas’ and evil spells – originally believed to have caused the Black Death and other widespread diseases as far back as we know – constitutes the understanding of microorganisms as the cause of infectious diseases. This insight, which dawned on medical researchers as late as the end of the 1800s, casts light on recurrent influenza pandemics and their strong association with the environment and worldwide travel. The recent transformation of SARS-CoV-2 (Severe acute respiratory syndrome coronavirus 2) COVID-19 from a local epidemic into a pandemic engulfing the whole world within months [6] is indeed a strong reminder of the relationship between contacts, host mobility and health.

Applying a functional perspective of urban centres, and proposing the location of new cities, the American architect Edward Ullman developed an architectural agenda that considered geographic, economic and sociologic issues, coining the term ‘transportation geography’ [7–9]. Transport plays a key role as it is strongly connected to access to water and sanitation as well as to healthcare facilities, and also influence disease control and global health. Spatial ecology and the genetic evolution of pathogens shape the dynamics of transmission [10]. Focusing on the intersection of evolutionary biology, epidemiology and geography (*i.e.* biogeography) can enable working towards an integrated view of spatial incidence, host mobility and viral genetic diversity, recognising that the translocation of pathogens is closely connected with the human role in transport of goods and commodities [11].

Public health was contemplated by the Swedish geographer Hägerstrand, who conceived the diffusion of an infectious disease across territories, or in populations, as a wave with an accelerating crescendo followed by a saturated slowing-down phase, hence the term ‘time geography’ was born [8, 12, 13]. This new view led to a swing from the previous emphasis on locality that had determined medical thinking since Hippocrates [14, 15] to a more systemic and eventual approach [16].

The association of environmental factors with human disease and survival is important, making spatio-temporal analysis particularly relevant for successful disease management and optimization of health care [17]. Health equity is closely connected to accessibility to healthcare and the targeting of healthcare resources (*i.e.* geolocation). This need for the geographically targeted approaches has led to interdisciplinary collaboration between public health and social sciences and between public health and spatial statistics, requiring connectivity and cross-linkage with global research [18]*.* This has led to integrated spatial surveillance systems that are indeed useful for tracking infection risk including the development of antimicrobial resistance (AMR) and incidence and exploration of environmental variables [19]*.*

### General information

**Fig. 1:**
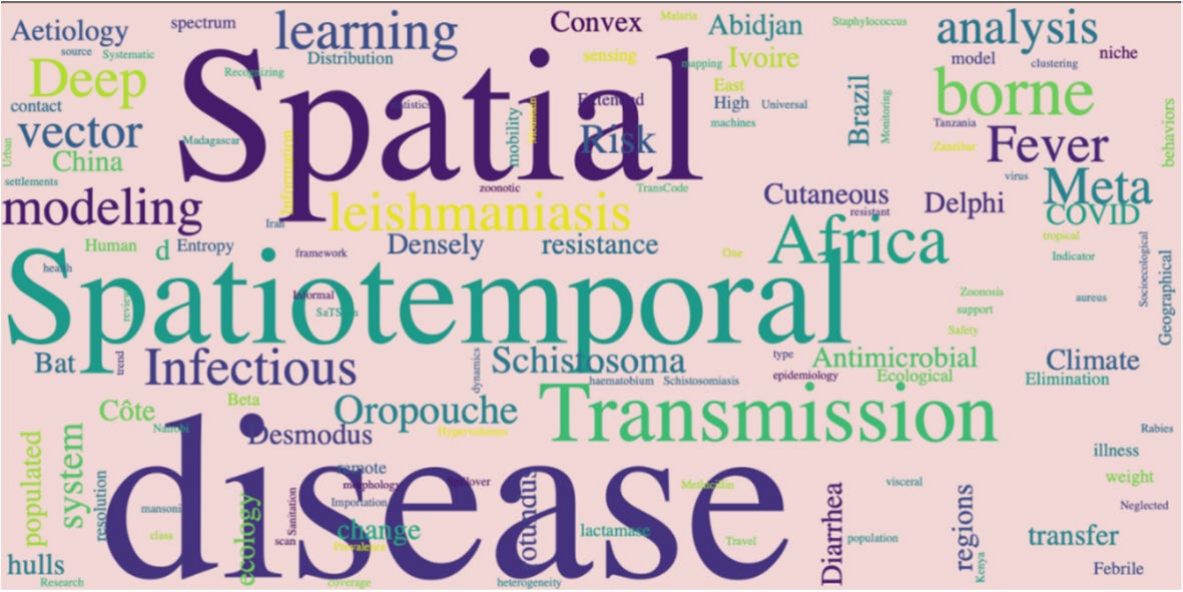
The keyword ensuing from the 14-article series covered in this special issue of *Infectious Diseases of Poverty*

The thematic series entitled “Geography and infectious diseases: role of human translocation and access to healthcare” was launched in late 2022. It attracted considerable interest, culminating in the 14 focal articles in *Infectious Diseases of Poverty*. This set of articles cover a large scientific field, ranging from translocation of infections by human movement to the implications of transport networks with emphasis on various aspects, such as the effect of expansion of urban and agricultural areas, deforestation, human-animal co-residence, zoonotic potential of multi-resistant bacteria, role of climatic change, invasion of exotic vector mosquitoes, emergence and re-emergence of infectious diseases. The interdisciplinary collaboration and spill-over into new hosts by zoonotic viruses. These 14 articles consist of eight research articles, three commentaries, two scoping reviews and one short report. The key words are presented in Fig. 1.

**Fig. 2:**
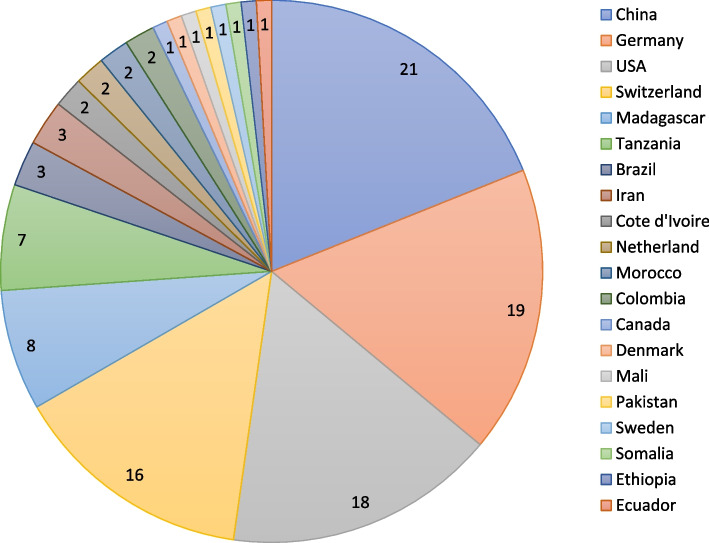
The country distribution of authors contributing to the 14 articles in the thematic series

The body of work concerns a total of 103 authors from 21 countries, including the People’s Republic of China (21 authors), Germany (19), United States of America (18), Switzerland (16), Madagascar (8), Tanzania (7), Brazil (3), Iran (3), Côte d'Ivoire (2), Colombia (2), Netherlands (2) and Morocco (2), followed by one author from each of Canada, Denmark, Ecuador, Ethiopia, Mali, Pakistan, Somalia and Sweden (Fig. 2).

**Fig. 3:**
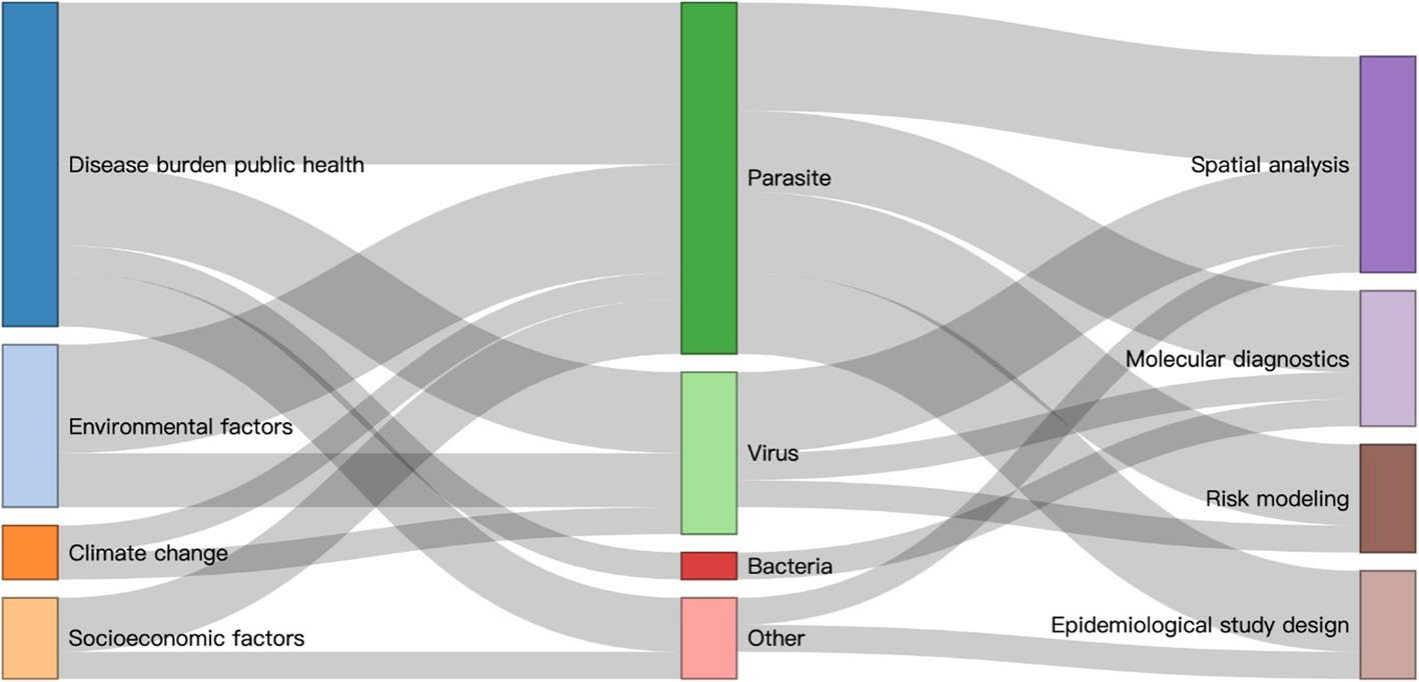
Sankey diagram showing the topics, pathogen types and approaches applied in the research presented

Figure 3 shows the topics, pathogen types and approaches used in this thematic series. Twelve articles focused on disease burdens with public health impact, six were on specific environmental factors, three described socioeconomic factors and two had emphasised on climate change. In terms of pathogens covered in this special issue, the majority focused on parasites (13 articles), six on viruses, one on bacteria and three on other pathogens. In terms of approaches, eight articles applied spatial analysis, five used molecular diagnostics, four applied risk modelling and four used epidemiological study designs.

### Key findings

#### Spatio-temporal surveillance on disease transmission patterns

Two articles address the spatial distribution of risk factors, in the context of surveillance Xue et al. [20] developed satellite-based models for recognition and monitoring livestock bovine as reservoir source of *Schistosoma japonicum* infection, demonstrating that high-resolution satellite images capturing the spatial distribution of bovine can be effective for surveillance, control and elimination of schistosomiasis in the People’s Republic of China. By means of a cross-sectional study in three specific regions of Madagascar, Gruninger et al. [21] focussed on the prevalence and risk distribution of schistosomiasis for adults living in endemic areas. Based on the general high level of infection, and the specific high risk for farmers, the results indicate the need to re-address control activities towards more context-specific, holistic and integrated approaches.

#### Assessment of disease transmission risks with environment factors

Two articles in the special issue deal with *Leishmania* spp., with Firouraghi et al*.* [22] investigating cutaneous leishmaniasis by spatio-temporal clinical and parasitological analysis, and Luo et al. [23] establishing a three-level indicator framework for assessing the risk of the mountain-type visceral leishmaniasis. Firouraghi et al*.* [22] detected high-risk zones and significant regional, temporal and spatio-temporal patterns in the distribution of leishmaniasis, showing the need to reduce socio-demographic, environmental factors, and sandfly (sandfly) reservoirs. Preventive issues relate to: (i) environmental and social indicators; (ii) climatic and geographical features; (iii) the sandfly vector and dog population density; and (iv) the importance of bed nets. Luo et al. [23] conducted two rounds of Delphi consultation identifying 4 primary indicators - including biological, environmental and social factors - further 11 secondary, 35 tertiary indicators.

Moreover, Magalhães et al. [24] expand on the role of natural ecosystems coupled with socioeconomic variables in the transmission probability of neglected tropical and zoonotic diseases in Brazil. The authors document the overall performance and relative variable importance between environmental and socioenvironmental model predictors of infectious disease presence. In order to efficiently address neglected tropical diseases, public health strategies must target both, reduction of poverty and cessation of destruction of natural forests and savannas.

#### Emerging or re-emerging diseases can be monitored by fever symptom surveillance

Nooh et al. [25] provide a meta-analysis of the prevalence of fever of unidentified aetiology in six East African countries. The authors emphasis a comprehensive fever surveillance to improve the differential diagnosis, to advance the outcome of treatment in countries with limited diagnostic tools and to identify multiple pathogens with higher fidelity. The most prevalent fever-causing pathogen was found to be Chikungunya virus, followed by *Plasmodium* spp. In a cross-sectional study on accessibility to healthcare in informal settlement of sub-Saharan Africa, Pessoa Colombo et al. [26] compared environmental determinants of access to shared sanitation in informal settlements in Cote d’Ivoire and Kenya. Location and security of sanitation facilities related to the risk of diarrhoea. Location of sanitation is a critical determinant of perceived security and has to be adequately addressed when building new facilities.

#### Association between human mobility and disease translocation

Three articles focus on the theme of human translocation dynamics and transmission of infectious agents and diseases. Using a cross-sectional approach, Fakih et al. [27] documented the importation and translocation of malaria from mainland Tanzania to Zanzibar Island, concluding that the low *Plasmodium* prevalence in Zanzibar is compromised by such importation and translocation. Furthermore, the authors speculate that the more aggressive malaria vector species *Anopheles stephensi* to have been carried to Zanzibar via human or cargo transport. By applying a deep transfer model in the seven densely populated areas in Hong Kong, Tokyo, New York City, San Francisco, Toronto, London and Berlin, Ren et al. [28] reveal heterogeneous fine-scale spatio-temporal transmission patterns of SARS-CoV-2 (COVID-19)*.* They argue that the heterogeneous patterns contribute to a heterogeneous spread of the virus requiring different intervention strategies. The main contributors to the rapid spread of the virus were found to be mobility and contact behaviours. Romero-Alvarez et al. [29] investigated the transmission risk of Oropouche fever in South-America, caused by a less established *Orthobunya* virus. They developed and applied spatial epidemiological models of the transmission risk, detecting hotspots in seven tropical Latin American countries leaving up to five million people at risk for this infection. Vegetation loss appeared to be the driver of this zoonotic vector-borne fever emergence.

#### Spill-over transmission becomes major avenue to increase the incidence and geographic range of zoonoses

Escobar et al. [30] focused on the effect of deforestation resulting in the transmission of viruses from an original or primary reservoir host to a novel, susceptible and permissive host species followed by successful onward intra-specific transmission putting humans at risk. Various factors can contribute to the likelihood of such spill-over transmission and successful virus establishment: (i) susceptibility (in the print version: (i) susceptibility in one line) of the novel host; (ii) immune status and genetics of humans at risk; (iii) population density; and (iv) host sex and age of the hosts. Concerning the regional and global dimension biodiversity, agriculture, livestock and climate change affect spill-over transmission. Future research needs to include landscape conditions, biodiversity, viral mutations and the important role of climate change. The rabies virus is primarily a pathogen in various carnivores inflicting heavy impact on humans and livestock throughout Latin America and elsewhere. Outbreaks from other spill-over transmissions in the last two decades include the swine acute diarrhoea syndrome originally caused by the transmission of an alpha-corona virus from bats to pigs in the People’s Republic of China and further: the Marburg virus from the African fruit bat to primates in Africa; the severe acute respiratory syndrome corona virus (SARS-CoV-1, a beta-corona virus) from bats to palm civets; and the Middle East respiratory syndrome (MERS-CoV), another beta-corona virus, from bats to camels in the Middle East. As a result of global climate and environmental change, spill-over transmission is expected to increase the incidence and enlarge the geographic range in the future.

#### An emerging risk caused by antimicrobial resistant bacterial infections

A highly relevant topic of infectious diseases and geography is addressed in a commentary by Olaru et al. [31]. Reservoirs and sources of AMR are not only found in animals and humans, but also in the environment. Livestock farming accounts for substantial use of antimicrobials eventually leading to drug resistance. Highly expressed contamination of extended-spectrum ß-lactamase-producing *Escherichia coli* is highly expressed in poultry and birds whom can disperse the resistant bacteria over large distances. The spread of drug-resistant bacteria is a specific problem in areas where sanitary systems are poor and coprophagous *Diptera* (also called “filth flies”) can act as vectors. In addition, humans are also exposed to resistant bacteria by meat from non-domesticated animals and food produced by aquaculture. AMR can be mediated e.g. by *E. coli* producing an extended-spectrum of ß-lactamase-positive antibiotic or by methicillin-resistant *Staphylococcus aureus*. In addition, AMR can spread by horizontal gene transfer implemented by plasmids, bacteriophages or by uptake of extracellular DNA. AMR can result through mutations of chromosomal or extra-chromosomal genes including acquisition of resistance genes from other organisms.

#### Research into the impacts of climate changes on infectious diseases are still overlooked

Two articles emphasise the role of climatic changes in transmission of pathogens and emergent infectious diseases. The scoping reviews by Van de Vuurst & Escobar [32] focused on vector-borne pathogens and conducted in high-income countries revealed taxonomic and geographic biases, both with regard to the type of disease transmission and to the localities where they occurred. The authors express that research in this area has failed to be socially inclusive, geographically balanced and sufficiently broad in terms of the disease systems as a whole. To this end, they recommend that future research on climate change and infectious diseases should have a broader focus to also consider diseases relying on direct, close contact to better understand the actual effects of climate change on health.

Lühken et al. [33] demonstrate that climate change and globalization increased the establishment of invasive mosquitoes, e.g. *Aedes albopictus* in Europe, and that the outbreak opportunities of infections by mosquito-borne viruses have increased worldwide. In their article, they thus promote the need for more control, research and monitoring.

### Gaps identified

The geography and spread of infectious diseases are indeed an area of growing relevance in an increasingly interconnected world, a fact that this thematic series wishes to draw attention to. Several areas in today’s surveillance and the response system with important links to geography and health have been identified. Urgent action is required with respect to:

1. insufficient diagnostic sensitivity in disease detection addressing biases in assessments, particularly concerning regional variations in disease occurrence and stratification of diagnostic determinants;
2. lack of recognition of re-infection patterns post-treatment restricts widespread utilization of diagnostics in disease detection and surveillance, raising concerns about the representativeness of the study population due to sampling methods;
3. limited utilization of remote sensing technology acquiring high-resolution earth observed imagery in disease prediction and early warning systems;
4. absence of consideration of the impacts of climate change on various diseases has limited the focus primarily to vector-borne pathogens and research conducted in high-income countries, neglecting social inclusivity and geographical diversity;
5. limited understanding of spill-over transmission mechanisms, which hampers the ability to predict this mode of transmission during the emergence of infectious diseases;
6. constraints in data sharing, particularly concerning morbidity, population mobility, social contacts, and healthcare information, which have introduced significant biases in measuring infection occurrences. This had led to underestimation of disease incidence; a restricted set of indicators for transmission risk assessment; limited exploration of local mobility; insufficient study of social contact patterns; and an inadequate understanding of the sylvatic cycle of diseases and wildlife reservoirs; as well as
7. slow enhancement of modelling techniques, since developments in this area are hindered by the absence of essential data.

### Recommendations

To mitigate the transmission risks of infectious diseases in the post-COVID-19 pandemic era, and to effectively address the identified gaps in the thematic series, the following recommendations are proposed:

- Improving the assessment protocols for potential diagnostic evaluations to address biases linked to regional variations in disease occurrence and stratification of diagnostic determinants is vital for enhancing diagnostic sensitivity in the field.
- Research endeavours on serum diagnostics to recognise re-infection patterns post-treatment, alongside meticulously planned sampling approaches, essential for ensuring enhanced representativeness of the study population.
- Enhancing diagnostic capabilities and surveillance-response systems through advanced fever symptom surveillance is critical for the early identification and response to emerging infectious diseases.
- Facilitating collaboration with remote-sensing management will mitigate topical and taxonomic biases through an ecosystem-based framework and its responses to climate change.
- Boosting research efforts to explore the impact of climate change on various disease transmissions, with specific focus on the emergence of vector-borne diseases in low- and middle-income countries, is important. It would intensify research on the health effects of sanitation interventions in diverse social-ecological settings and contribute to evaluating the circulation of viruses across different species, regions and landscapes to proactive prevention measures associated with vegetation loss.
- Advancing research on the spill-over transmission mechanisms of emerging zoonotic diseases is vital for improving our capacity to respond promptly to such occurrences, and could effectively prevent importation and clearance of infections by focusing on identified high-risk groups.
- Developing strategies to monitor and control AMR, along with optimizing antimicrobial usage in low- and middle-income countries, would help combat global health threat.
- Promoting multi-sectoral collaboration in data sharing and coordination, coupled with advancing research to enhance the management of datasets for accurate and timely disease surveillance, would support the prediction and management of outbreaks of emerging diseases when integrated with real-time data.
- Aligning public health strategies with poverty alleviation and ecosystem conservation policies by promoting health policies that emphasise specific, holistic and integrated control strategies should sustainably reduce morbidity.
- Evaluating the influence of sociodemographic factors, environmental elements and vector reservoirs in the emergence and re-emergence of zoonotic diseases should contribute to intensifying research on the health effects of sanitation interventions in diverse social-ecological contexts.

## Data Availability

The data have been published in 14 articles in *Infectious Diseases of Poverty* reviewed in the article and cited in the references**.**

## Abbreviations

AMR: Antimicrobial resistance
BioClim: Bioclimatic variables
MERS-CoV: Middle East respiratory syndrome coronavirus
SARS-CoV-1: Severe acute respiratory syndrome coronavirus 1
SARS-CoV-2: Severe acute respiratory syndrome coronavirus 2
WHO: World Health Organization
WorldClim: Global climate data

## References

[1] Pavlovskii EN (1945). The ecological parasitology. J Gen Biol.

[2] Hutchinson GE (1957). Concluding remarks. Cold Spring Harbor Sympos Quantitat Biol.

[3] Hijman RJ, Cameron SE, Parra JL, Jones PG, Jarvis A (2005). Very high resolution interpolated climate surfaces for global land areas. Int J Climatol.

[4] Booth TH, Nix HA, Busby JR, Hutchinson MF (2014). Bioclim: the first species distribution modelling package, its early applications and relevance to most current MaxEnt studies. Biodivers Rev Divers Distrib.

[5] Winslow CE (1920). The untilled field of public health. Mod Med.

[6] Bergquist R, Rinaldi L (2020). Covid-19: pandemonium in our time. Geospat Health.

[7] Ullman EL. Transportation geography. Office of Naval Research. Contract no. Nonr-477(03), Report No. 9. University of Washington, Seattle WA, USA 1954. https://apps.dtic.mil/sti/tr/pdf/AD0034733.pdf

[8] Amer S, Bergquist R. Transport geography: implication for public health. Geospat Health. 2021;16(1):1–2. 10.4081/gh.2021.1009. 10.4081/gh.2021.1009 33969969

[9] Murray KA, Olivero J, Roche B, Tiedt S, Guégan JF. Pathogeography: leveraging the biogeography (this word without word division) of human infectious diseases for global health management. Ecogeography. 2018;41(9):1411–1427. 10.1111/ecog.03625. 10.1111/ecog.03625 PMC7163494 32313369

[10] Woolhouse MEJ, Webster JP, Domingo E, Charlesworth B, Levin BR. Biological and biomedical implications (this word without division) of the co-evolution of pathogens and their hosts. Nature Genetics. 2002;32:569–77. 10.1038/ng1202-569. 10.1038/ng1202-569 12457190

[11] Townsend Peterson A (2008). Biogeography of diseases: a framework for analysis. Naturwissenschaften.

[12] Cliff AD, Haggett P, Smallman-Raynor M (2008). An exploratory method for estimating the changing speed of epidemic waves from historical data. Int J Epidemiol.

[13] Schaerström A. Disease diffusion. International Encyclopedia Human Geography. 2009;222–233. 10.1016/B978-008044910-4.00330-8.

[14] Hippocrates. On airs, waters, and places. Written ca 400 BC and translated by Francis Adams. Available from: http://classics.mit.edu/Hippocrates/airwatpl.1.1.html.

[15] Suvajdžić L, Đendić A, Sakač V, Čanak G, Dankuc D (2016). Hippocrates – The father of modern medicine. Vojnosanit Pregl.

[16] Wang W, Cao Y (2022). Network diversity and health change among international migrants in China: evidence from foreigners in Changchun. Int J Environ Res Public Health.

[17] Eisenberg JN, Desai MA, Levy K, Bates SJ, Liang S, Naumoff K (2007). Environmental determinants of infectious disease: a framework for tracking causal links and guiding public health research. Environ Health Perspect.

[18] Hierink F, Okiro EA, Flahault A, Ray N (2021). The winding road to health: a systematic scoping review on the effect of geographical accessibility to health care on infectious diseases in low- and middle-income countries. PLoS One.

[19] Pitout JDD. Transmission surveillance for antimicrobial-resistant organisms in the health system. MicrobiolSpectr. 2018;6(5). doi: 10.1128/microbiolspec. MTBP-0010-2016. 10.1128/microbiolspec.mtbp-0010-2016 PMC11633628 30191805

[20] Xue JB, Xia S, Wang XY, Huang LL, Huang LY, Hao YW, Zhang LJ, Li SZ. Recognizing and monitoring infectious sources of schistosomiasis by developing deep learning models with high-resolution remote sensing images. Infect Dis Poverty. 2023;12:6–18. 10.1186/s40249-023-01060-9. 10.1186/s40249-023-01060-9 PMC9903608 36747280

[21] Gruninger SK, Rasamoelina T, Rakotoarivelo RA, Razafindrakoto AR, Rasolojaona ZT, Rakotozafy RM (2023). Prevalence and risk distribution of schistosomiasis among adults in Madagascar: a cross-sectional study. Infect Dis Poverty.

[22] Firouraghi N, Bergquist R, Fatima M, Mohammadi A, Hamer DH, Shirzadi MR (2023). High-risk spatiotemporal patterns of cutaneous leishmaniasis: a nationwide study in Iran from 2011 to 2020. Infect Dis Poverty.

[23] Luo Z, Zhou Z, Hao Y, Feng J, Gong Y, Li Y (2022). Establishment of an indicator framework for the transmission risk of the mountain-type zoonotic visceral leishmaniasis based on the Delphi-entropy weight method. Infect Dis Poverty.

[24] Magalhães AR, Codeço CT, Svenning JC, Escobar LE, Van de Vuurst P, Gonçalves-Souza T (2023). Neglected tropical diseases risk correlates with poverty and early ecosystem destruction. Infect Dis Poverty.

[25] Nooh F, Chernet A, Reither K, Okuma J, Brattig NW, Utzinger J (2023). Prevalence of fever of unidentified aetiology in East African adolescents and adults: a systematic review and meta-analysis. Infect Dis Poverty.

[26] Pessoa Colombo V, Chenal J, Orina F, Meme H, Koffi JDA, Koné B (2023). Environmental determinants of access to shared sanitation in informal settlements: a cross-sectional study in Abidjan and Nairobi. Infect Dis Poverty.

[27] Fakih BS, Holzschuh A, Ross A, Stuck L, Abdul R, Al-Mafazy AH (2023). Risk of imported malaria infections in Zanzibar: a cross-sectional study. Infect Dis Poverty.

[28] Ren J, Liu Y, Liu J (2023). TransCode: Uncovering COVID-19 transmission patterns via deep learning. Infec Dis Poverty.

[29] Romero-Alvarez D, Escobar LE, Auguste AJ, Del Valle SY, Manore CA (2023). Transmission risk of *Oropouche* fever across the Americas. Infect Dis Poverty.

[30] Escobar L, Velasco-Villa A, Satheshkumar P, Nakazawa Y, Van de Vuurst. Revealing the complexity of vampire bat rabies spillover transmission. Infect Dis Poverty. 2023;12:10–9. 10.1186/s40249-023-01062-7. 10.1186/s40249-023-01062-7 PMC9924873 36782311

[31] Olaru ID, Walther B, Schaumburg F (2023). Zoonotic sources and the spread of antimicrobial resistance from the perspective of low and middle-income countries. Infect Dis Poverty.

[32] Van de Vuurst P, Escobar LE (2023). Climate change and infectious disease: a review of evidence and research trends. Infect Dis Poverty..

[33] Luehken R, Brattig N, Becker N. Introduction of invasive species into Europe and prospects for arbovirus transmission and vectior control in an era of globalization. Infect Dis Poverty. 2023;12(1):109–24. 10.1186/s40249-023-01167-z. 10.1186/s40249-023-01167-z PMC10687857 38037192

